# Supplementary material for: Dissecting the spatiotemporal diversity of adult neural stem cells
Source: Mol Syst Biol. 2024 Feb 16;20(4):321–37. doi: 10.1038/s44320-024-00022-z (PMC10987636; doi:10.1038/s44320-024-00022-z)
Supplement: Supplementary file 1 — Appendix [file 44320_2024_22_MOESM1_ESM.pdf]

Appendix:

Appendix Figure S1 : Marker genes for subtypes of radial glia (related to Fig. 1) - page 2
Appendix Figure S2: Comparison to mouse disease models (related to Fig. 1) - page 3
Appendix Figure S3: Spatial origin of scRNAseq libraries (related to Fig. 2) - pages 4, 5
Appendix Figure S4: Additional RNAscope data (related to Fig. 1 and 2) - pages 6,7
Appendix Figure S5: Technical information and replicate experiment for lineage tracing (related to Fig. 3) - page 8
Appendix Figure S6: Terminal states in the telencephalon, and cell types involved in neurogenesis in other brain regions (related to Fig. 4) - pages 9,10
Appendix Figure S7: Quality control of scSLAM-seq datasets (related to Fig. 5) - page 11

Appendix Figure S1

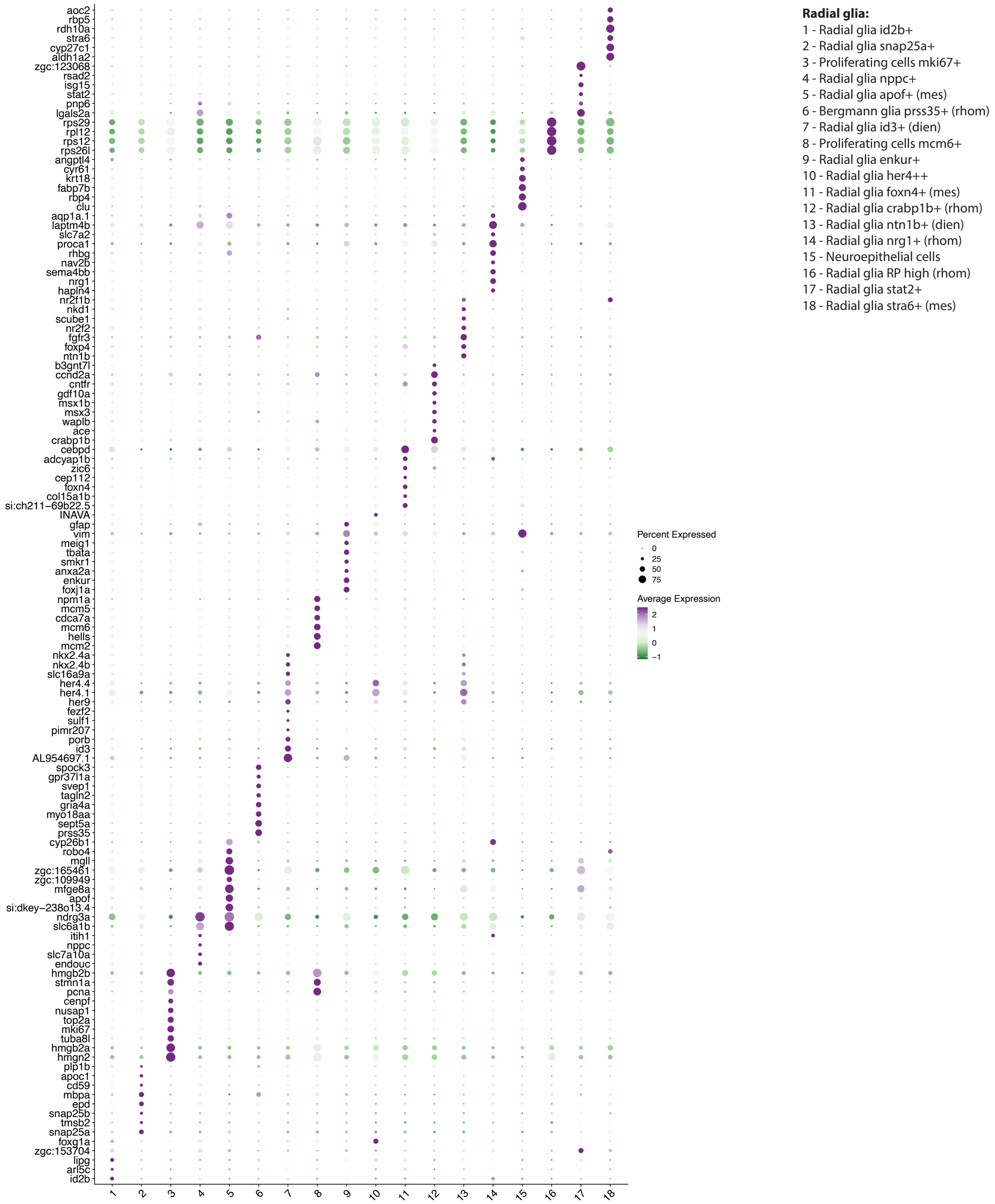

Appendix Figure S1. Marker genes for subtypes of radial glia (related to Fig. 1)
Expression of marker genes in radial glia subtypes. A total of 15,829 cells were grouped into 18 clusters, with several representative marker genes shown for each cluster. The diameter of the dot indicates the fraction of cells of that cluster that express that gene, while the color intensity denotes the average expression value (based on scaled data). Sample IDs b1 - b20 were used for this analysis.

Appendix Figure S2

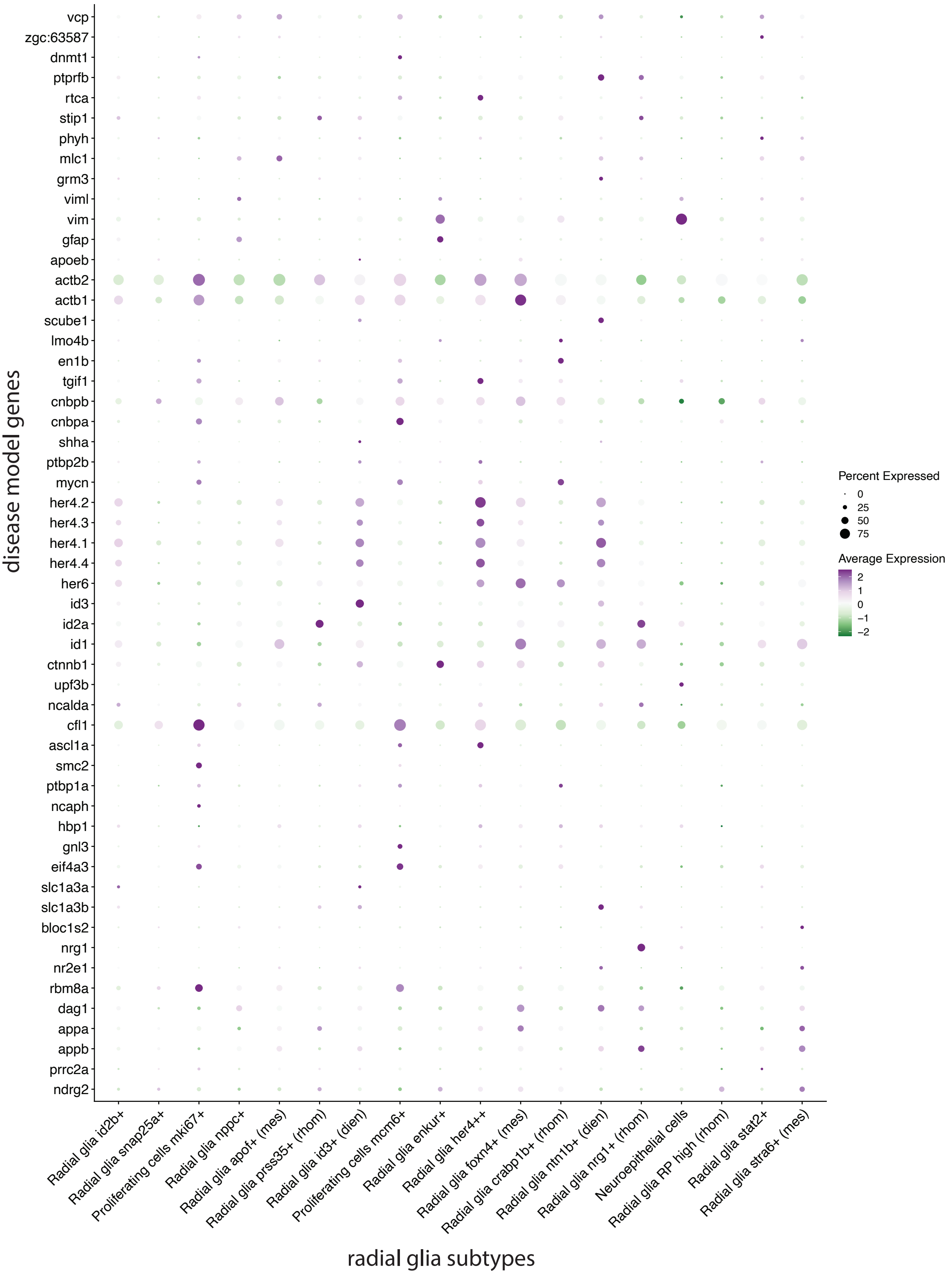

Appendix Figure S2. Comparison to mouse disease models (related to Fig. 1).
Expression of genes that are implicated in specific diseases or abnormalities of the nervous system (obtained from the Mouse Genome Informatics database) in radial glia subtypes (see Methods).

Appendix Figure S3 - page 1

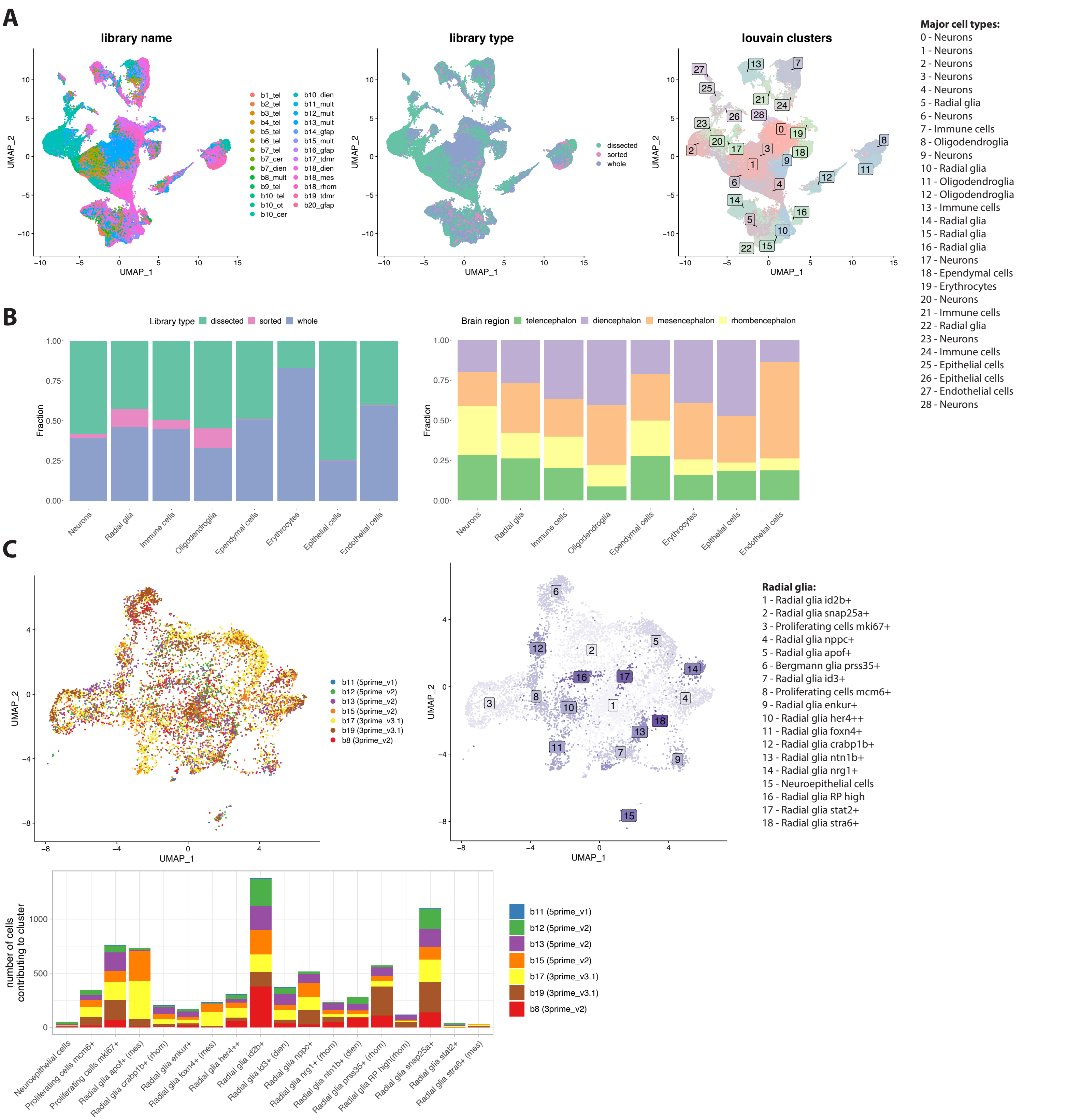

Appendix Figure S3. Spatial origin of scRNAseq libraries (related to Fig. 2)

A – UMAP representations of the composition of the complete scRNA-seq dataset of the adult zebrafish brain (107598 single cells, sample IDs b1- b20). Left: individual libraries (total of 27 libraries from 20 biological samples). Middle: library type, indicating whether the library was prepared from a dissected brain region, the whole brain, or sorted cells from the whole brain of transgenic gfap:GFP fish followed by FACS isolation. All sorted cells were used for downstream analysis, including sorted non-radial glia cells.

Right: Louvain clusters, with the assigned cell type identity indicated in the text box. See EV 2 for marker genes for each cluster.

B – Bar plots depicting the library type (left) or spatial origin (right) of each of the major cell types. The library type plot shows fraction of library type per cell type, for the spatial origin plot, only cells that come from dissected libraries (sample IDs b1-b7, b9, b10 and b18) were taken into account and the data was normalized by brain region.

C – Batch effect analysis of radial glia cells. Only whole brain, non-FACSed samples were included for which the expectation (under ideal experimental conditions) would be equal representation of all cell states. The versions of the 10x Genomics Chromium kit used for each library are listed, since this would be a potential source of batch effects. Top row: UMAP representation of radial glia, color represents sample ID and library type (left) or cluster (right). Bottom row: Bar graph representing samples contributing to each cluster. Each radial glia subtype contains cells from multiple libraries, suggesting that clustering of subtypes is not dominated by batch effects.

Appendix Figure S3 - page 2

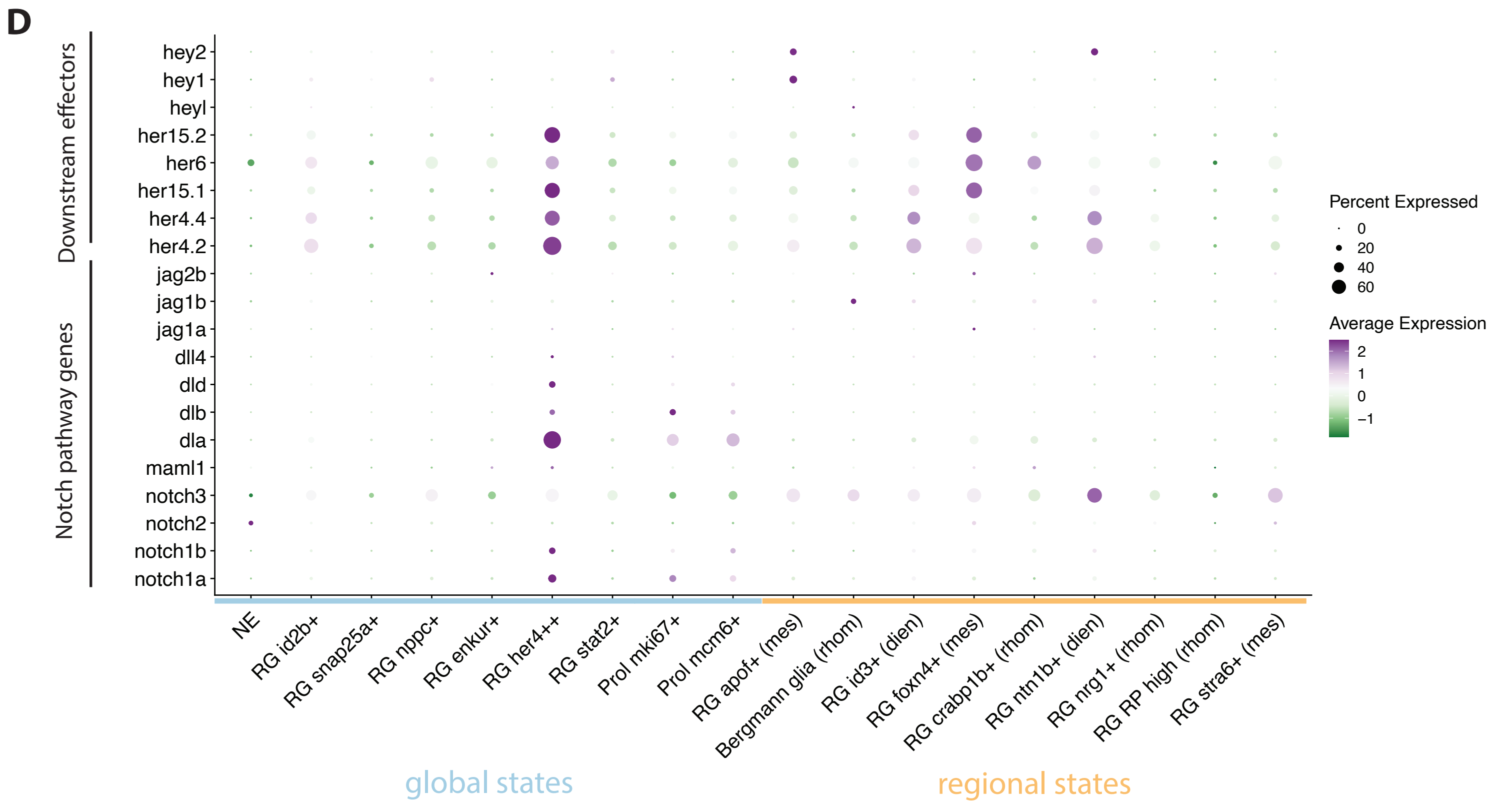

D – Expression of individual genes active in the Notch pathway or downstream Notch effectors expressed in radial glia subtypes (aggregate gene module score shown in Fig. 2B, sample IDs b1 - b20 used for this analysis).

Appendix Figure S4, page 1

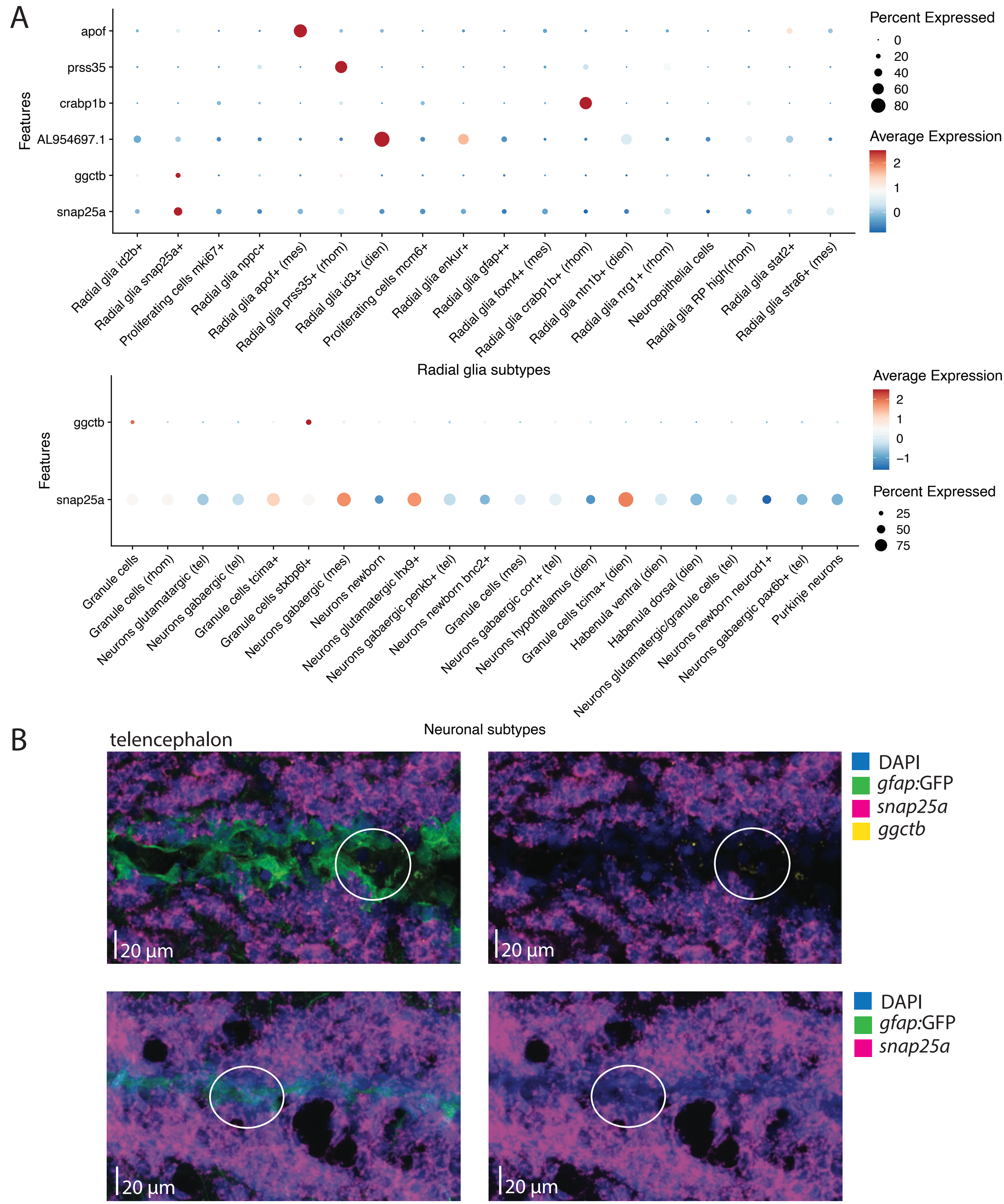

Additional RNAscope data:

A – dot blot showing expression of radial glia marker genes and marker genes of snap25+ RG in neuronal subtypes. Sample IDs b1 - b20 were used for this figure.

B – Medial sections of the telencephalon. Nuclei stained in blue using DAPI, radial glia cells in green (*gfap*:GFP). Presence of snap25+ RG markers *snap25a* (both rows) as well as *ggctb* (upper row only) shown using RNAscope probes. Images on the left have green channel hidden for clarity. Circes highlight areas of co-localization of *gfap* and *snap25a* (both rows) as well as *ggctb* (upper row only).

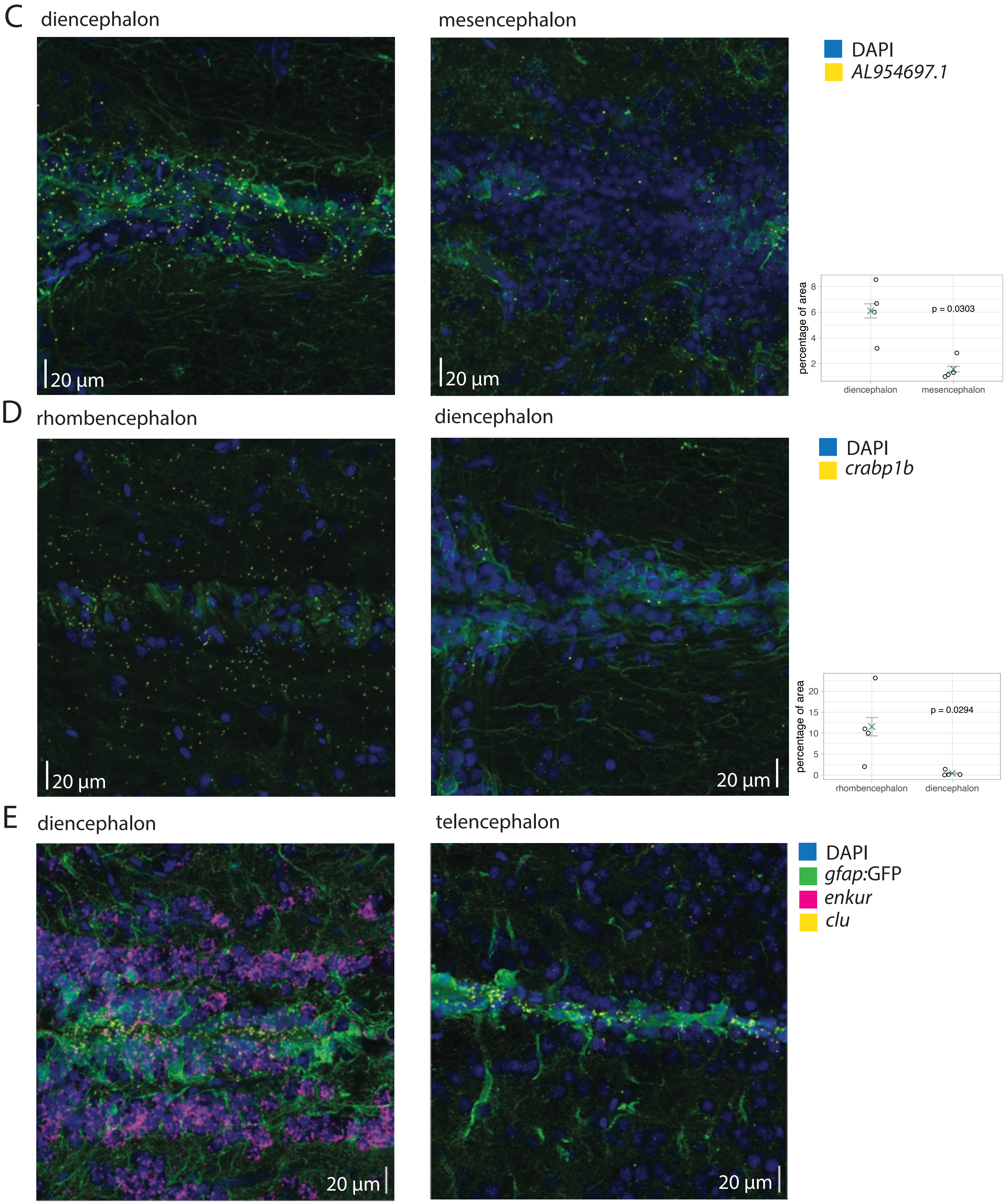

Additional RNAscope data:

C – medial sections of diencephalon and mesencephalon. Blue: Nuclei (DAPI), green: radial glia (*gfap*:GFP), yellow: *AL954697.1* (RNAscope probe); D – medial sections of rhombencephalon and diencephalon. Blue: Nuclei (DAPI), green: radial glia (*gfap*:GFP), yellow: *crabp1b* (RNAscope probe); Quantification of C and D using Fiji software: The RNAscope signal was measured in the section area containing radial glia cells, as determined based on the gfap:GFP signal. The Region of Interest was applied to the thresholded RNAscope signal, and the fraction of the ROI covered by the RNAscope signal (Fraction of Area) was measured. In the plots, each dot represents one single animal (one representative section per animal). Significance was tested using Mann-Whitney test, p-value is indicated on the plot. Error bars depict SEM, green crosses indicate mean.

E – medial sections of diencephalon and telencephalon, taken from the areas lining the ventricle. Blue: Nuclei (DAPI), green: radial glia (*gfap*:GFP), yellow: *clu* (RNAscope probe), magenta: *enkur* (RNAscope probe)

Appendix Figure S5

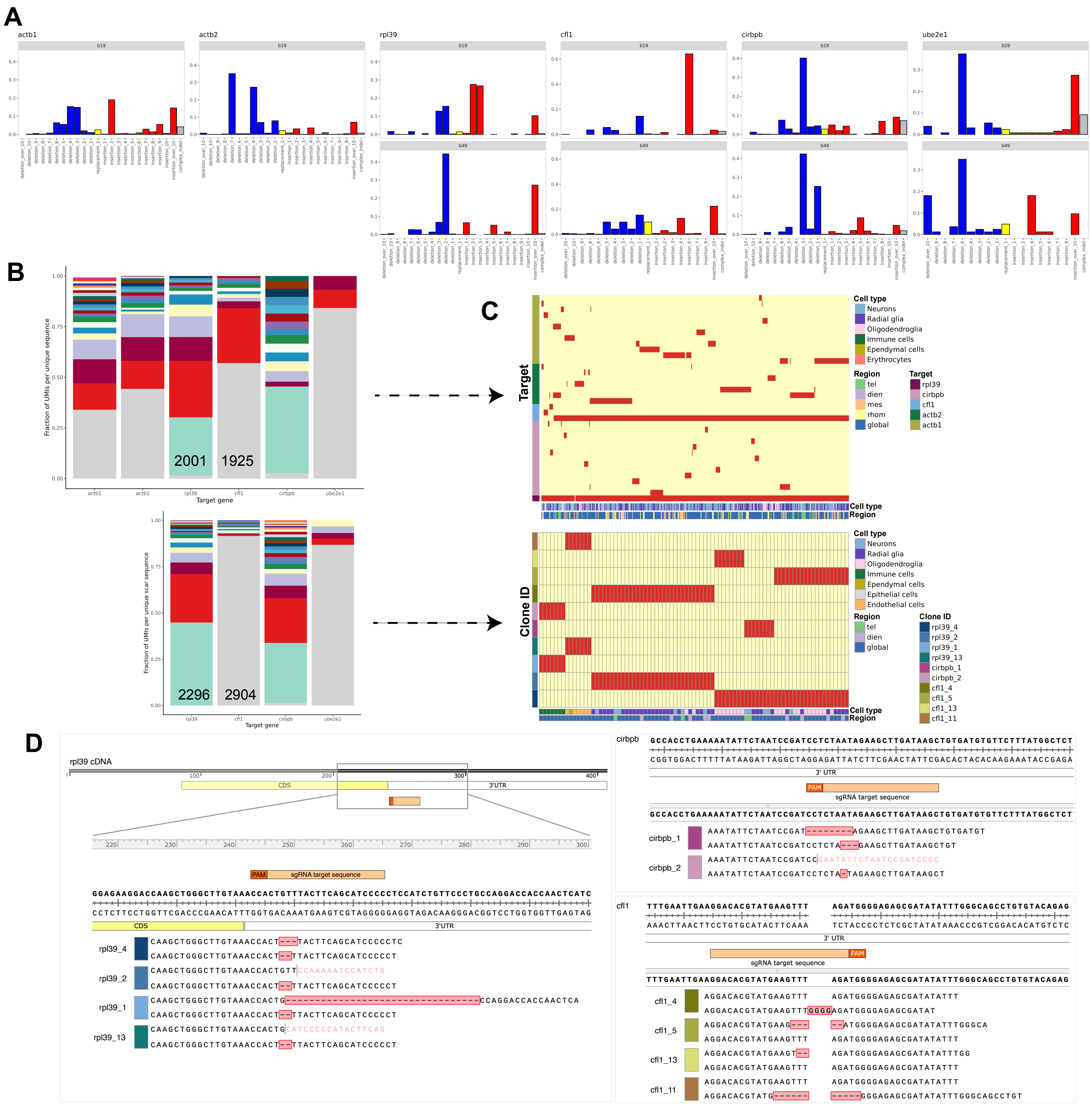

Appendix Figure S5. Technical information and replicate experiment for lineage tracing (related to Fig. 3), libraries: lintrace_rep1 & rep2
A – The lineage barcodes (or "scars") are the result of an imperfect repair mechanism following a double-stranded break of DNA. The types of lineage barcodes that are most commonly created and their abundances are represented in this plot separated by dataset (top panel: replicate 1 that was included in Fig. 3C, bottom panel: replicate 2) and targeted gene (individual bar plots). Among the types of edits we can differentiate between three main categories as indicated by the color of the bar: deletions (blue), substitutions (yellow) and insertions (red). The values on the x-axis represent the base pair length of the edited sequence and the y-axis the frequency of the sequence in the filtered transcripts across all cells of the dataset. B – Bar plot depicting the relative abundance of different sequences per target for each dataset (top panel: replicate 1, bottom panel: replicate 2). The values on the y-axis represent the fraction of transcripts (UMIs) recovered for each unique sequence (color slice within the bar), while the x-axis shows the targeted endogenous gene. The values at the bottom of the bar show the number of cells for which this particular target was efficiently recovered and which contribute to the fraction score. C – The heatmap shows results of hierarchical clustering of all cell (sub)types based on lineage barcodes for the two datasets (top panel: replicate 1, bottom panel: replicate 2). The identified clones are separated by targeted endogenous gene (top panel) or clone ID (bottom panel) indicated in the color bar on the y-axis, while the cell (sub)type and region of origin are indicated in the color bars on the x-axis. All cell types are included for both replicates (in contrast to Fig. 3C, which shows only radial glia and neurons for replicate 1). As expected, lineage clustering revealed a separation of neuronal cells and immune cells (see bottom panel), which validates the approach. D – Lineage barcode sequences defining clone IDs shown in C (bottom panel) aligned to the wildtype target sequence. The CRISPR/Cas9 target sites are located in the 3' UTR of endogenous genes, as shown for rpl39 on the left. Each clone ID is defined by two 40 base-pair sequence IDs, corresponding to the two alleles of the gene. These are shown for all clone IDs from C (bottom panel) as alignments to the wildtype sequence, indicating deletions or insertions.

Appendix Figure S6, page 1

A

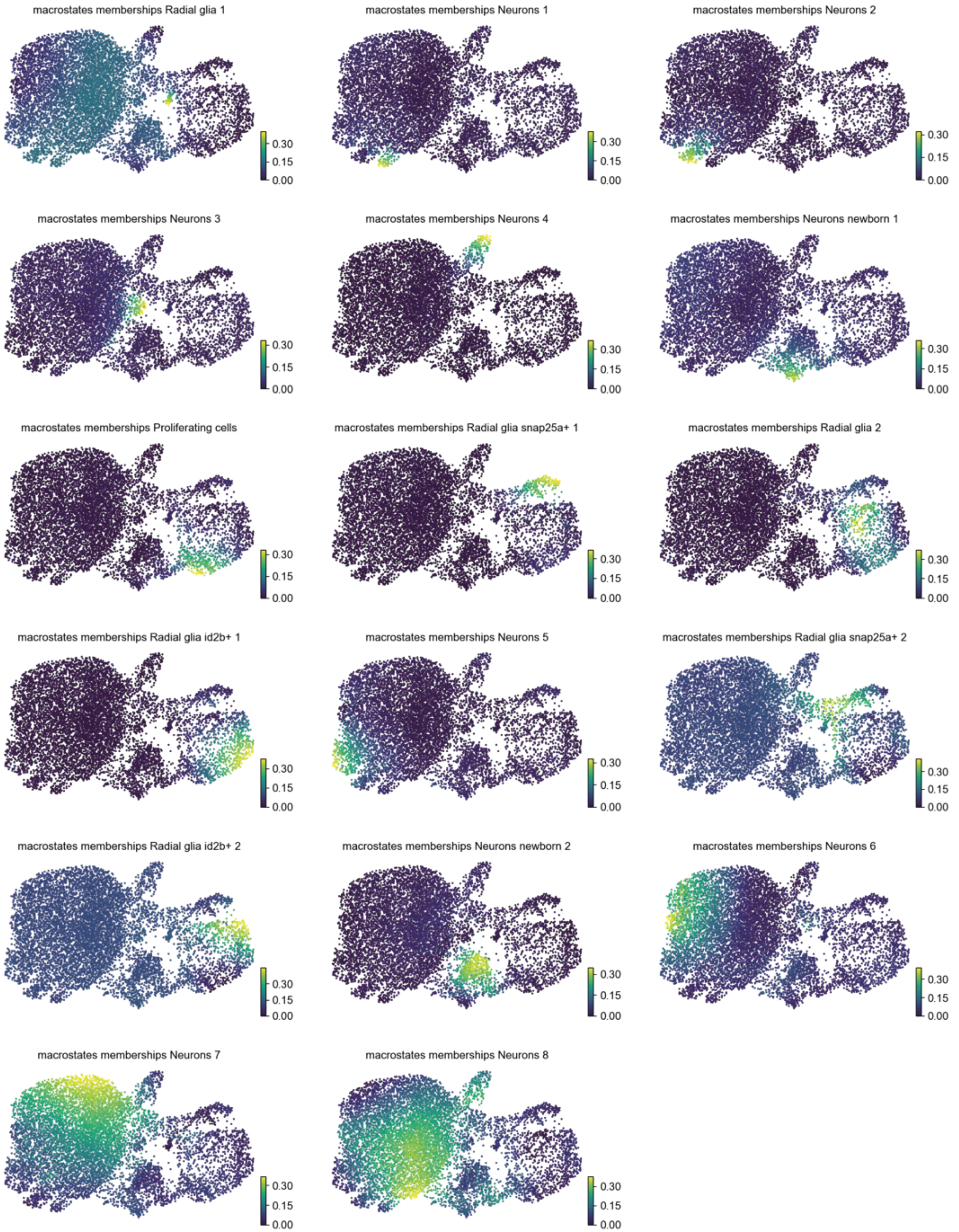

Appendix Figure S6. Terminal states in the telencephalon, and cell types involved in neurogenesis in other brain regions (related to Fig. 4)
A – Terminal states identified in the telencephalon by the CellRank algorithm (see Methods). These states were used to calculated absorption probabilities shown in Fig. 4C. Sample IDs b1 - b7 were used for this analysis.

Appendix Figure S6, page 2

B

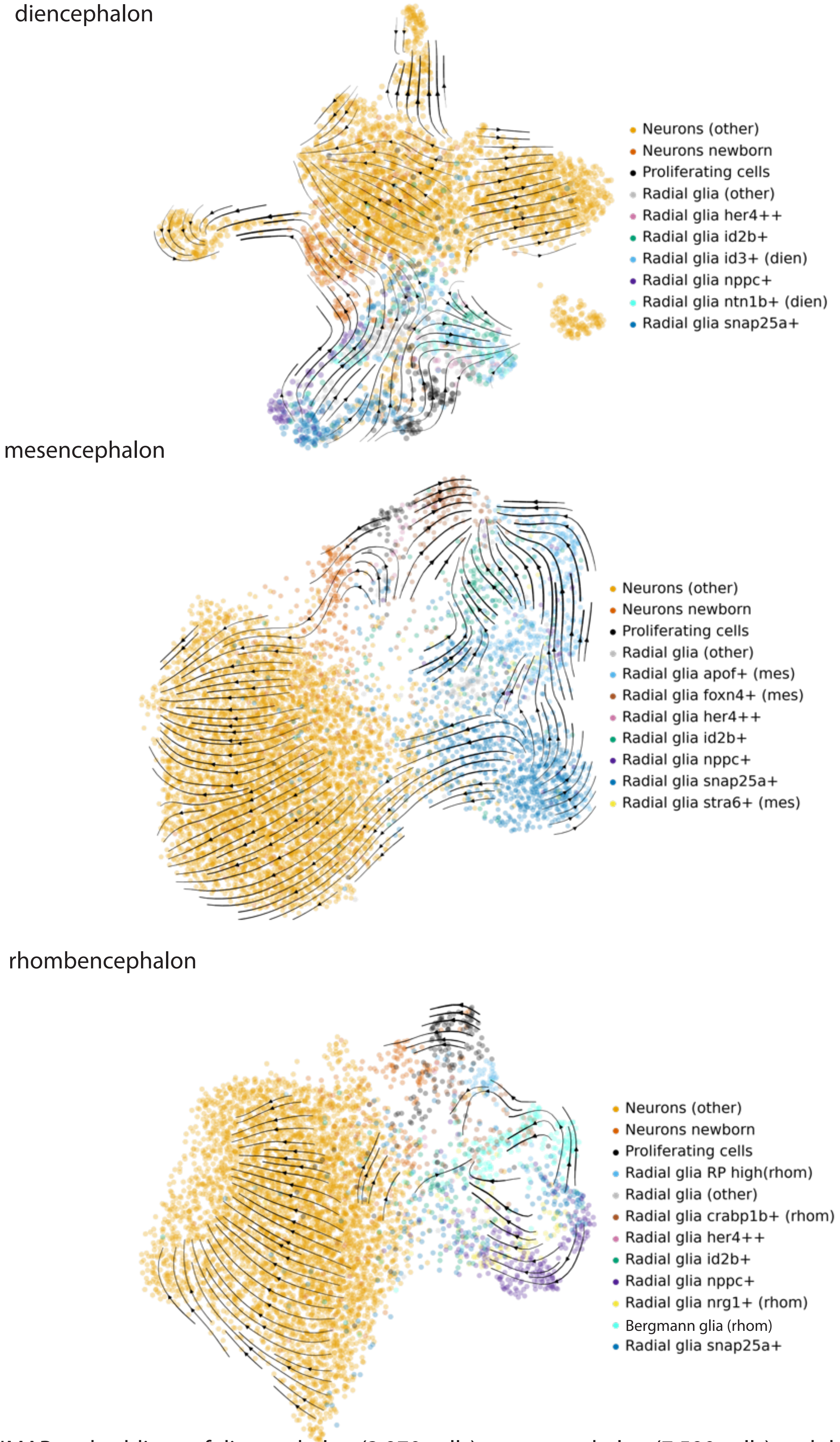

B – UMAP embeddings of diencephalon (3,970 cells), mesencephalon (7,590 cells) and rhombencephalon (5,917 cells). The highlighted subtypes within radial glia and neurons are the most abundant cell types identified in each region. For clarity, all cell states present with very low frequency within the brain region are classified as "Radial glia (other)" or "Neurons (other)". Sample ID b18 was used for this figure.

Appendix Figure S7

A Notch downstream genes expression - pseudobulk

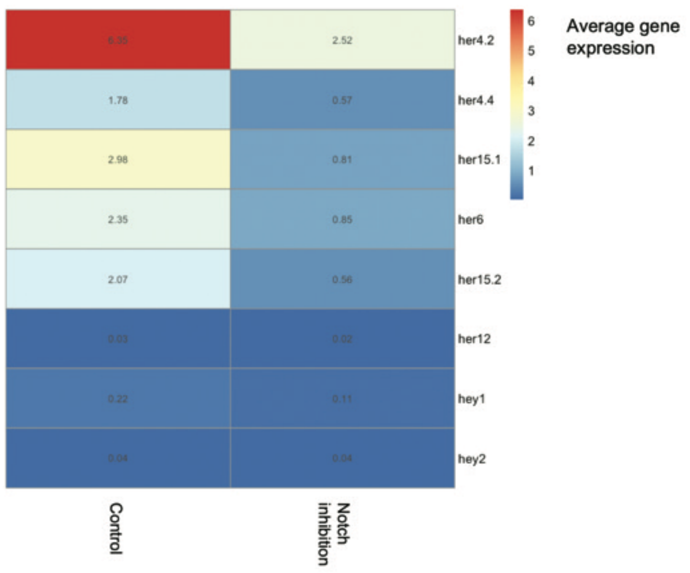

B

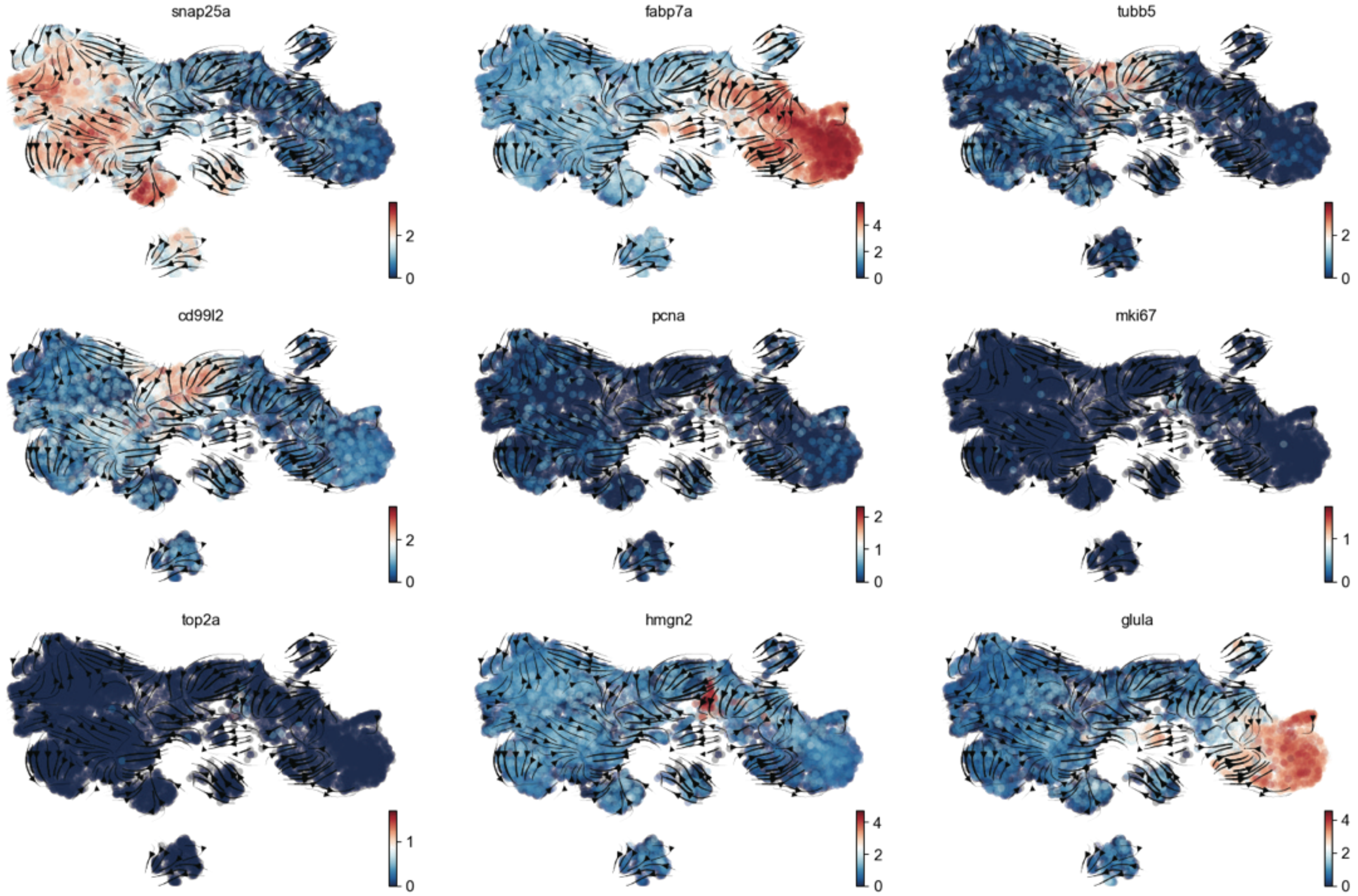

Appendix Figure S7. Quality control of scSLAM-seq datasets (related to Fig. 5), sample IDs b22 & b23
A – Heat map of average expression of Notch pathway genes in all radial glia clusters (based on log-normalized expression data) in the control (DMSO) and Notch inhibition (DAPT) datasets.
B – UMAP plot of combined scSLAM-seq dataset (as depicted in Fig. 5C) highlighting expression of individual marker genes representative for the defined cell states of interest. Based on the expression of marker genes in Leiden clusters, cells were broadly classified as "Radial glia" (fabp7a+) or "Neurons" (snap25a+). Furthermore, within radial glia two groups of cells were classified as "Radial glia snap25a" (fabp7a+ and snap25a+ co-expression) and "Proliferating radial glia" (pcna+, mki67+, top2a+, hmgn2+ and fabp7a+), while within neurons we additionally highlight "Newborn neurons" (snap25a+ and tubb5+).
